# Analysis of a large choroideremia dataset does not suggest a preference for inclusion of certain genotypes in future trials of gene therapy

**DOI:** 10.1002/mgg3.208

**Published:** 2016-02-28

**Authors:** Paul R. Freund, Yuri V. Sergeev, Ian M. MacDonald

**Affiliations:** ^1^ Department of Ophthalmology and Visual SciencesUniversity of AlbertaEdmontonAlbertaCanada; ^2^ Ophthalmic Genetics and Visual Function BranchNational Eye InstituteNational Institutes of HealthBethesdaMaryland

**Keywords:** Choroideremia, natural history, Rab escort protein‐1, retinal dystrophy, visual acuity, visual fields

## Abstract

**Background:**

Choroideremia (CHM) is an X‐linked degeneration of the retinal pigment epithelium, photoreceptors, and choroid, which causes nyctalopia and progressive constriction of visual fields leading to blindness. The *CHM* gene encodes Rab escort protein 1 (REP‐1). In this work, we reviewed the phenotypes and genotypes of affected males with the purpose of understanding the functional effects of *CHM* mutations and their relationship with the phenotypes.

**Methods:**

A retrospective review of 128 affected males was performed analyzing the onset of symptoms, visual acuity, and visual fields with respect to their mutations in the *CHM* gene.

**Results:**

In rank order, reflecting data from this report, the most common mutations found in the *CHM* gene were nonsense mutations (41%), exon deletions (37%), and splice sites (14%) associated with a loss of functional protein. In the pool of 106 CHM mutations, we discovered four novel missense mutations (c.238C>T; p.L80F, c.819G>T; p.Q273H, c.1327A>G; p.M443V, and c.1370C>T; p.L457P) predicted to be severe changes affecting protein stability and folding with the effect similar to that of other types of mutations. No significant genotype–phenotype correlation was found with respect to the onset of nyctalopia, the onset of other visual symptoms, visual acuity, or width of visual fields.

**Conclusion:**

There is no evidence to support exclusion of CHM patients from clinical trials based on their genotypes or any potential genotype–phenotype correlations.

## Introduction

Choroideremia (CHM; MIM# 303100) is an X‐linked inherited retinal dystrophy characterized by degeneration of photoreceptors, retinal pigment epithelium, and ultimately, the choroid. Affected males develop nyctalopia, progressive loss of peripheral visual fields, and loss of central visual acuity in the late stage of the disease. The prevalence of CHM is estimated to be 1/50,000–1/100,000 (MacDonald et al. 1993). CHM is only known to be caused by mutations in the *CHM* gene affecting the expression of Rab escort protein 1 (REP‐1), which is an important part of the pathways that enable the prenylation of Rab proteins that are essential for intracellular trafficking (Cremers et al. 1990b; Merry et al. 1992). There are 136 reported pathogenic variants in the *CHM* gene associated with the CHM phenotype (Leiden Open Variant CHM Database, http://www.lovd.nl/CHM [accessed June 2015]).

To date, descriptions of the natural history of CHM have been significantly limited in scope by issues such as small sample sizes in case series, lack of confirmation of *CHM* mutations (Karna 1986), or by only describing changes in visual acuity (Roberts et al. 2002; Coussa et al. 2012). An extensive analysis of CHM phenotypes and a comparison with their genotypes has not been undertaken to date. In a cross‐sectional analysis, we chose to include the onset of symptoms, and patients' visual acuity and visual fields at specific ages as variables reflecting the severity of the disorder. As this is a progressive disorder, an affected male would be expected to have a decline in visual acuity and a restriction in visual field with increasing age. If there was a significant genotype–phenotype correlation, we would expect that to be reflected in the severity of the disorder.

As gene therapy trials for CHM are undertaken (http://www.ClinicalTrials.gov #NCT01461213, #NCT02077361, and #NCT02341807), we need accurate descriptions of the anticipated change in visual function over time observed in a CHM patient population with confirmed *CHM* mutations. Knowledge of the CHM phenotype and any correlation with genotype may influence the selection of subjects for inclusion in clinical trials, as well as the expected effects from ocular gene therapy on progression. For example, if a subject with a milder phenotype, predicted by the type of mutation, was included in a trial, the time course to monitor a change might be protracted. This individual might then not be selected in a Phase 2 trial designed to show efficacy. Ocular gene therapy trials are generally designed to measure an intended positive effect within a one to two year time frame; researchers, patients, funding agencies, and patient advocacy groups are anxious to move these initial experiments to potential treatments. Our analysis of a large dataset of genotypes and phenotypes of CHM patients was intended to search for any preferences for the inclusion (or exclusion) of patients in future clinical trials of gene therapy.

## Materials and Methods

### Ethical compliance

This study received research ethics approval from the University of Alberta Health Research Ethics Board and all procedures conformed to the Code of Ethics of the World Medical Association (Declaration of Helsinki). Written informed consent was obtained from all individuals included in the databases.

### Mutation analysis

Genotyping was performed on blood samples submitted to the University of Alberta or eyeGENE^™^ program (MacDonald et al. 2004). The 15 exons of the *CHM* gene (GenBank NM_000390.2) and their immediately adjacent intron sequences were sequenced.

In order to search for any preferences for the inclusion of patients with missense mutations in future clinical trials of gene therapy, the severity of protein perturbations caused by novel missense changes was evaluated. For this purpose, the amino acid sequences of Rab proteins, geranylgeranyl transferase component A1, and REP‐1 (RAE1_HUMAN) were retrieved from the UniProtKB database (http://www.uniprot.org/uniprot/P35556). The crystal structures were recovered from the protein database (http://www.rcsb.org/pdb/home/home.do) for the REP‐1 protein in a complex with monoprenylated Rab7 protein (PDB file: 1vg0_A, 481 residues with quality score 0.498) and the structure of REP‐1 in a complex with Rab geranylgeranyl transferase and isoprenoid (PDB file: 1LTX‐R, 494 residues with quality score 0.374) and used as structural templates. The human REP‐1 structure was built by an automated protein‐homology modeling module incorporated in a molecular visualization, modeling, and dynamics program, YASARA (Krieger et al. 2004, 2006) (www.yasara.org). The hybrid model of the human REP‐1 obtained with Z‐score −0.945 was generated, refined, and 26 ns equilibrated in water using the Amber03 force field and periodic boundary conditions. Protein stabilization energy changes, ∆∆G_mut_, the propensity of mutant variant to be in unfolded state, U, were evaluated for each mutant variant, p.Leu80Phe, p.Gly273His, p.Met443Val, p.Leu457Pro, p.Leu550Pro, and (p.Leu80Phe; p.Met443Val), using a semiempirical method for calculating free energies (Schymkowitz et al. 2005). The structure of the protein complex, which includes human REP‐1, Rab7, and Rab GGTase‐2, was generated similar to that of described by Sergeev et al. using rat structures as structural templates (PDBfiles: 1vg0, 1ltx). Several online prediction programs were also used to assess the missense mutations (details available in Supporting Information, Data S1).

### Databases

Novel *CHM* variants described in this study have been submitted to the Leiden Open Variant CHM Database (http://www.lovd.nl/CHM) curated by David Baux. Variants that were extracted from the eyeGENE^™^ database are also registered in the eyeGENE‐curated Leiden Open Variant Database for CHM (https://grenada.lumc.nl/LOVD2/NIH/eyegene/home.php?select_db=CHM).

### Clinical data

Clinical data were accessed from patient databases at the University of Alberta, Edmonton, Canada and eyeGENE^™^, at the National Eye Institute of the National Institutes of Health, Bethesda, MD. One hundred and twenty‐eight males with a clinical diagnosis of CHM and confirmed *CHM* mutations were included in the study. Fifty‐nine males were from the University of Alberta database and sixty‐nine males were from the eyeGENE^™^ database. Clinical data were submitted to the databases by subjects' ophthalmologists via a standardized form collecting subjects' history and clinical exam. The age of onset of symptoms (nyctalopia and other symptoms of visual loss) and any pertinent family history were recorded. Best‐corrected visual acuities (BCVAs) were converted to logMAR notation. Semiquantitative BCVAs were converted to logMAR equivalents as follows: counting fingers (CF) = 1.9, hand motion (HM) = 2.3 (Schulze‐Bonsel et al. 2006). Light perception vision (LP) was assigned a logMAR value of 2.7 and no light perception (NLP) was assigned a value of 3.0. To best describe the residual visual function, the eye with better BCVA was included for analysis. The demographic and clinical features of the CHM subjects are summarized in Table 1.

**Table 1:** Demographic and clinical features of subjects

	Age group (years)	1–10	11–20	21–30	31–40	41–50	51–60	61–70	71–80	Total
Best‐corrected visual acuity^a^	*n*	10	28	17	19	24	15	12	3	128
	Age (mean ± SD)	8.3 ± 1.2	16.0 ± 2.7	25.5 ± 2.8	36.0 ± 2.9	46.0 ± 2.7	54.6 ± 2.3	65.2 ± 3.1	72.7 ± 1.2	35.7 ± 18.5
	Age (median)	9	17	26	37	46	55	64	72	36
	VA (mean ± SD)	0.075 ± 0.118	0.045 ± 0.126	0.016 ± 0.059	0.099 ± 0.172	0.372 ± 0.497	0.818 ± 0.765	0.848 ± 0.845	2.567 ± 0.231	0.338 ± 0.630
	VA (median)	0.05	0	0	0	0.3	0.5	0.439	2.7	0.1
	Age group (years)	1–10	11–20	21–30	31–40	41–50	51–60	61–70	71–80	Total
Visual field width^b^	*n*	5	16	7	7	15	10	4	0	64
	Age (mean ± SD)	8.8 ± 0.5	15.6 ± 2.6	25.9 ± 2.5	35.3 ± 3.1	45.9 ± 2.6	54.3 ± 2.9	65.0 ± 2.8		34.6 ± 17.6
	Age (median)	9	15.5	26	37	46	54.5	64		37
	VF (mean ± SD)	73 ± 51	74 ± 56	42 ± 45	19 ± 11	13 ± 9	6 ± 6	4 ± 2		35 ± 45
	VF (median)	60	50	25	15	12	5	4		20

a Visual acuities are in logMAR units. The best‐corrected visual acuity of the better eye is used.

b Visual fields are defined as the width of the continuous visual field across the horizontal meridian (in degrees) using a Goldmann perimeter with the III4e isopter. The visual field of the better eye is used.

VA, visual acuity; VF, visual field; SD, standard deviation.

Visual fields (VFs) were quantified by the width of the continuous VF across the horizontal meridian (in degrees) using a Goldmann perimeter (Haag‐Streit AG, Koeniz, Switzerland) with the III4e isopter. The physiological blindspot was not considered a break in the VF if the central field was continuous with the more peripheral field. VFs not performed with the III4e isopter were excluded. In the absence of Goldmann perimetry results, central VFs quantified with the Humphrey Field Analyzer (stimulus size III; Carl Zeiss, Dublin, CA) or MAIA microperimeter (Centervue, Padova, Italy) was used. The eye with the better VF was included in the analysis.

Electroretinographic recordings (ERG), performed according to standards outlined by the International Society for Clinical Electrophysiology of Vision (Marmor et al. 2009) were collected in the databases. Stimulator, software, and electrodes used were decided according to the testing laboratory. Dark‐adapted mixed responses and light‐adapted flicker responses were analyzed. The presence or absence of fundus changes (retinal atrophy, choroidal atrophy, or vascular attenuation) was also recorded.

### Statistics

For statistical analyses, the threshold of *P* < 0.05 was used to reject the null hypotheses. Post hoc tests were corrected with the Bonferroni method to account for multiple comparisons. Statistical analysis was performed with Prism 6 (GraphPad Software Inc., La Jolla, CA) and SPSS 20 (IBM Corp., Armonk, NY). Subjects were grouped by decade (at the age of the subject's most recent assessment) and inclusion/exclusion tests were performed to define the critical age for changes in VA and VF (Coussa et al. 2012). Briefly, subjects were grouped by decade and Kruskall–Wallis ANOVAs were performed with Dunn's correction for multiple comparisons to detect differences between the groups. If there was no significant difference between the youngest group and the second‐youngest group, the two groups were combined and the procedure was repeated. When the pooled group was significantly different from the next oldest group, that age was defined as the critical age. The subgroups defined by the critical age were analyzed with linear regression modeling with age as the predictor variable.

The paucity of missense mutations and large number of unique *CHM* mutations in our sample precludes the use of mutations as a predictor variable in the linear regression models. To identify any potential genotype–phenotype correlations, data from individuals with either missense mutations or mutations causing a lack of any REP‐1 expression (due to whole gene deletions or deletions of the ATG start codon) were highlighted and qualitatively compared.

## Results

### CHM mutations

**Figure 1:**
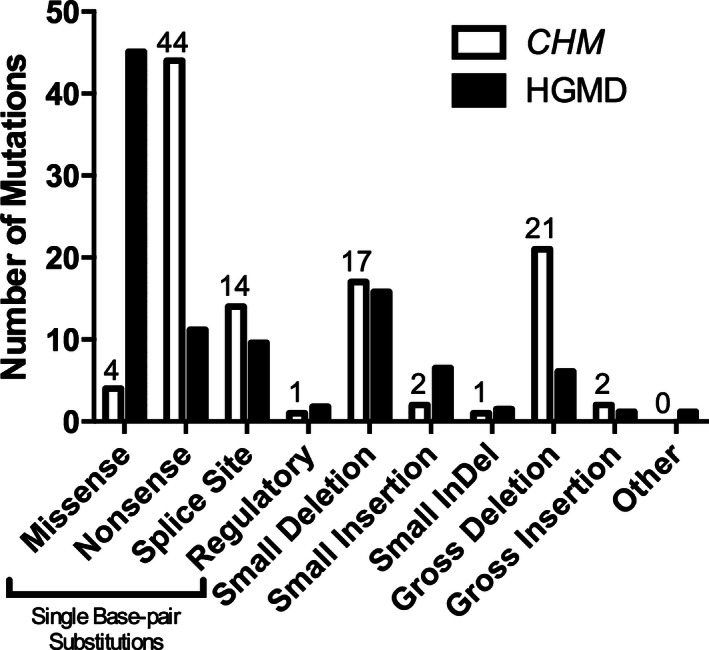
Proportion of *CHM* (MIM# 303100) gene mutations observed in 106 affected families compared to the mutations reported in the Human Genome Mutation Database (HGMD).

The mutations of each family are described in Table 2. There were 66 different mutations detected in 106 different families. In comparison to the mutations recorded in the Human Genome Mutation Database (HGMD) (Stenson et al. 2009), *CHM* mutations have a significantly different distribution (Fig. 1; *χ* ^2^ = 133.4, df = 9, *P* < 0.001). Mutations in *CHM* are more frequently nonsense or frameshift mutations resulting in premature truncation, with a paucity of missense mutations compared to the rest of the genome. Within this dataset, mutations in 22 families were associated with complete gene or exon deletion. Two families carried exon duplications. Frameshift and splice site mutations were found in 21 and 10 families, respectively. Five missense mutations were detected in four families (4/106; 3.8%), of which four mutations had not previously been described: (c.819G>T (p.Gln273His); c.1370T>C (p.Leu457Pro); and one family had two missense mutations: c.238C>T (p.Leu80Phe) and c.1327A>G (p.Met443Val). The fifth mutation (c.1649T>C [p.Leu550Pro]) has been previously described in detail (Sergeev et al. 2009).

**Table 2:** *CHM* a mutations

Family	Mutation	Exon	Protein change	Previous reports
1	c.‐29‐?_*3450+?del	1–15	Complete gene deletion – REP‐1 absent	Cremers et al. (1990a)
2	c.‐29‐?_*3450+?del	1–15	Complete gene deletion – REP‐1 absent	
3	c.‐29‐?_*3450+?del	1–15	Complete gene deletion – REP‐1 absent	
4	c.‐29‐?_*3450+?del	1–15	Complete gene deletion – REP‐1 absent	
5	c.‐29‐?_*3450+?del	1–15	Complete gene deletion – REP‐1 absent	
6	c.‐29‐?_*3450+?del	1–15	Complete gene deletion – REP‐1 absent	
7	c.‐29‐?_*3450+?del	1–15	Complete gene deletion – REP‐1 absent	
8	c.‐29‐?_*3450+?del	1–15	Complete gene deletion – REP‐1 absent	
9	c.‐29‐?_*3450+?del	1–15	Complete gene deletion – REP‐1 absent	
10	c.‐29‐(57‐63kb)_49+?del	1	Deletion of exon 1 – REP‐1 absent	van den Hurk et al. (2003) and McTaggart et al. (2002)
11	c.‐29‐(57‐63kb)_49+?del	1	Deletion of exon 1 – REP‐1 absent	
12	c.‐29‐(57‐63kb)_49+?del	1	Deletion of exon 1 – REP‐1 absent	
13	c.‐29‐?_49+?del	1	Deletion of exon 1 – REP‐1 absent	
14	c.3G>A	1	Affects start codon – REP‐1 absent	Strunnikova et al. (2009)
15	c.25_28delTTTG insAGTAATAGTAA	1	p.Phe9Serfs*14	
16	c.37delG	1	p.Val13*	
17	c.49+1G>A	intron 1	Splice site mutation	McTaggart et al. (2002)
18	c.49+3A>G	intron 1	Splice site mutation	
19	c.49+3A>G	intron 1	Splice site mutation	
20	c.49+3A>G	intron 1	Splice site mutation	
21	c.50‐?_314+?del	2–4	Deletion of exons 2–4 – (out‐of‐frame)	
22	c.116+1G>T	intron 2	p.Gly17Glufs*21	Esposito et al. (2011)
23	c.116+1G>C	intron 2	Splice site mutation	
24	c.116+1G>C	intron 2	Splice site mutation	
25	c.116+1G>C	intron 2	Splice site mutation	
26	c.117‐?_1166+?dup	3–8	Duplication of exons 3–8 – unable to predict	Chi et al. (2012)
27	c.117‐?_1510+?dup	3–12	Duplication of exons 3–12 – unable to predict	
28	c.167dupT	3	p.Leu56Phefs*12	
29	c.190‐2A>G	intron 3	Splice site mutation – p.Glu64*	van den Hurk et al. (2003)
30	c.225G>A	4	p.Trp75*	
31	c.232C>T	4	p.Gln78*	
32	c.315‐?_1166+?del	5–8	Deletion of exons 5–8 – (in‐frame) – REP‐1 absent	McTaggart et al. (2002)
33	c.316C>T	5	p.Gln106*	Nesslinger et al. (1996)
34	c.470_473delAAAC	5	p.Gln157Leufs*10	
35	c.525_526delAG	5	p.Glu177Lysfs*6	van Bokhoven et al. (1994)
36	c.525_526delAG	5	p.Glu177Lysfs*6	
37	c.525_526delAG	5	p.Glu177Lysfs*6	
38	c.563_564delTG	5	p.Val188Alafs*10	MacDonald et al. (2004)
39	c.564_565delGC	5	p.Pro189Ilefs*11	Fujiki et al. (1999)
40	c.652_655delTCAC	5	p.Ser218Lysfs*13	McTaggart et al. (2002)
41	c.653C>G	5	p.Ser218*	
42	c.700A>T	5	p.Lys234*	Strunnikova et al. (2009)
43	c.700A>T	5	p.Lys234*	
44	c.703‐?_940+?del	6–7	Deletion of exons 6–7 – p.Leu235Argfs*4	Strunnikova et al. (2009) and Esposito et al. (2011)
45	c.703‐?_940+?del	6–7	Deletion of exons 6–7 – p.Leu235Argfs*4	
46	c.703‐?_940+?del	6–7	Deletion of exons 6–7 – p.Leu235Argfs*4	
47	c.703‐?_1166+?del	6–8	Deletion of exons 6–8 – (out‐of‐frame)	
48	c.715C>T	6	p.Arg239*	McTaggart et al. (2002)
49	c.715C>T	6	p.Arg239*	
50	c.757C>T	6	p.Arg253*	Fujiki et al. (1999)
51	c.757C>T	6	p.Arg253*	
52	c.757C>T	6	p.Arg253*	
53	c.757C>T	6	p.Arg253*	
54	c.757C>T	6	p.Arg253*	
55	c.757C>T	6	p.Arg253*	
56	c.757C>T	6	p.Arg253*	
57	c.757C>T	6	p.Arg253*	
58	c.757C>T	6	p.Arg253*	
59	c.757C>T	6	p.Arg253*	
60	c.799C>T	6	p.Arg267*	van den Hurk et al. (1997)
61	c.799C>T	6	p.Arg267*	
62	c.799C>T	6	p.Arg267*	
63	c.799C>T	6	p.Arg267*	
64	c.799C>T	6	p.Arg267*	
65	c.808C>T	6	p.Arg270*	Fujiki et al. (1999)
66	c.808C>T	6	p.Arg270*	
67	c.808C>T	6	p.Arg270*	
68	c.817C>T	6	p.Gln273*	Strunnikova et al. (2009)
69	c.819G>T	6	p.Gln273His	
70	c.820‐1G>C	intron 6	Splice site mutation – skips exon 7, absent REP‐1	Potter et al. (2004)
71	c.846delT; c.881G>T	7	p.Phe282Leufs*9 (c.881 is beyond the premature stop codon)	
72	c.877C>T	7	p.Arg293*	van Bokhoven et al. (1994)
73	c.877C>T	7	p.Arg293*	
74	c.885_886insA	7	p.Met296Asnfs*11	
75	c.889A>T	7	p.Lys297*	
76	c.894delT	7	p.Thr300Hisfs*25	
77	c.910G>T	7	p.Glu304*	
78	c.993delC	8	p.Asn332Thrfs*12	
79	c.1034C>G	8	p.Ser345*	Fujiki et al. (1999)
80	c.1167‐?_1413+?del	9–11	Deletion of exons 9–11 – (out‐of‐frame)	
81	c.1184delG	9	p.Gly395Valfs*14	
82	c.1194T>G	9	p.Tyr398*	Huang et al. (2012)
83	c.1218C>A	9	p.Cys406*	Nesslinger et al. (1996)
84	c.1218C>A	9	p.Cys406*	
85	c.1234G>T	9	p.Glu412*	
86	c.1273C>T	10	p.Gln425*	
87	c.1327_1328delAT	10	p.Met443Valfs*18	Strunnikova et al. (2009)
88	c.1350‐6T>G	intron 10	Skip exon 11 – p.Arg450Argfs*4^b^	McTaggart et al. (2002)
89	c.1350‐2A>C	intron 10	Splice site mutation	Renner et al. (2009)
90	c.1350‐(14–10)delTTGT	intron 10	Small deletion affecting a splice site	
91	c.1363delG	11	p.Ala455Glnfs*3	Nesslinger et al. (1996)
92	c.1370T>C	11	p.Leu457Pro	
93	c.1511‐?_1609+?del	13	Deletion of exon 13 – (in‐frame)	
94	c.1511‐1G>A	intron 12	Splice site mutation	
95	c.1511‐1G>A	intron 12	Splice site mutation	
96	c.1512T>A	13	p.Tyr504*	McTaggart et al. (2002)
97	c.1584_1587delTGTT	13	p.Val529Hisfs*7	van den Hurk et al. (1992)
98	c.1603G>T	13	p.Glu535*	McTaggart et al. (2002)
99	c.1624G>T	14	p.Glu542*	
100	c.1624delG	14	p.Glu542Leufs*13	Jacobson et al. (2006)
101	c.1649T>C	14	p.Leu550Pro	Sergeev et al. (2009)
102	C.1670C>A	14	p.Ser557*	
103	C.1670C>A	14	p.Ser557*	
104	c.1670C>A	14	p.Ser557*	
105	c.1697_1698delAT	14	p.Asn566Argfs*19	
106^b^	c.238C>T;	4	p.Leu80Phe	Bentley et al. (2008)
	c.1327A>G	10	p.Met443Val	

a *CHM* GenBank reference sequence NM_000390.2.

b Two *CHM* mutations were present in this family.

Analysis of the effect of missense changes presents a challenge. In our work, we used the atomic structure of protein to predict *in silico* the effect of missense changes on protein structure. In order to evaluate the severity of missense changes, we significantly improved the previously published structure of human REP‐1 (Sergeev et al. 2009), refined this model with 26 ns molecular dynamics, and evaluated changes in free protein stabilization energy due to the missense change. From the analysis at the level of protein atomic structures, the missense variants, p.Gln273His, p.Leu457Pro, Leu550Pro, and p.Leu80Phe and p.Met443Val double mutation show a significant loss of protein stability possibly associated with a high level of protein unfolding (Table 3). The expression of both p.Met443Val and p.Leu550Pro mutations demonstrates an additive effect in decreasing the protein stability free changes, which is reflected in the predicted unfolding propensity.

**Table 3:** Protein stability in REP‐1 missense mutations

Mutation	∆∆G	U	F
p.Leu80Phe	0.467	0.685	0.315
p.Gln273His	1.482	0.922	0.078
p.Met443Val	6.423	1.000	0.000
p.Leu457Pro	8.475	1.000	0.000
p.Leu550Pro	7.539	1.000	0.000
p.Leu80Phe and p.Met443Val	5.795	1.000	0.000

Protein stability free energy changes (∆∆G) and unfolding (U)/folding (F) propensities for missense variants favors the REP‐1 unfolding.

**Figure 2:**
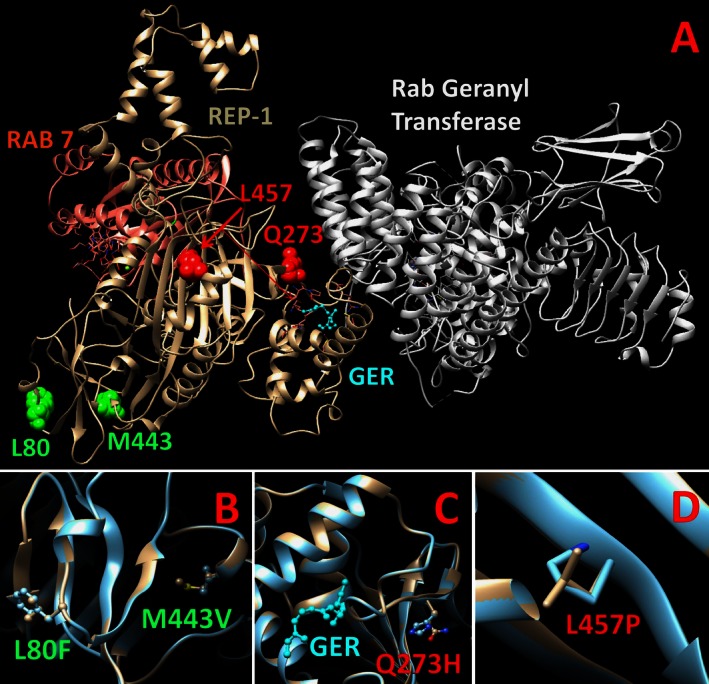
The structure of Rab escort protein 1 (REP‐1; beige) in relation to Rab geranyl transferase (white) and Ras‐associated protein 7 (RAB 7; pink) and its substrate geranylgeranyl (GER; blue). The REP‐1 double mutant, L80F and M443V, is highlighted in panel B (green); the Q273H (red) and L457P (red) mutations are in panels C and D, respectively.

These results were confirmed by several classification programs that evaluated the novel missense mutations (summarized in Table 4). The p.Gln273His substitution is predicted to be damaging to protein function by 5/7 prediction models (if normal mRNA splicing were to occur despite alteration of the splice site). This substitution is located within the pocket that holds geranylgeranylpyrophosphate and would affect the stability of substrate binding (Fig. 2). All (7/7) of the models predict that the c.1370C>T (p.Leu457Pro) mutation has a significant effect on the REP‐1 protein. The p.Leu457Pro substitution is located within a *β*‐sheet in domain 1 of REP‐1, responsible for its association with Rab protein substrates. The two variants (c.238C>T and c.1327A>G) that occurred in one family are both predicted to potentially alter splicing through alteration and creation of splicing enhancers and suppressors. The p.Leu80Phe and p.Met443Val substitutions do not change the hydrophobic property of residues in positions 80 and 443, which are located within *α*‐helixes in domain 1 of REP‐1. These two substitutions are both predicted to be neutral by 6/7 models. However, these models only consider these mutations in isolation compared to our protein structure model which modeled p.Leu80Phe and p.Met443Val together and predicted that REP‐1 is more unstable when both mutations are expressed. In summary, the *in silico* analysis suggests that the effect of missense changes appear to be similar to the effect of “null” mutations causing the absence of functional protein.

**Table 4:** Missense mutation prediction models

		c.238C>T	c.819G>T	c.1327A>G	c.1370C>T
		p.Leu80Phe	p.Gln273His	p.Met443Val	p.Leu457Pro
PolyPhen‐2 HumVar	Effect	Neutral	**Probably damaging**	Neutral	**Probably damaging**
	Score	0.442	**0.951**	0	**0.99**
SNAP	Effect	Neutral	Neutral	Neutral	**Nonneutral**
	Accuracy	60%	53%	53%	**63%**
pMUT	Effect	Neutral	Neutral	**Pathological**	**Pathological**
	Output	0.0646	0.2309	**0.5116**	**0.9169**
PROVEAN	Effect	Neutral	**Deleterious**	Neutral	**Deleterious**
	Score	−1.104	**−3.343**	−0.295	**−5.484**
SIFT	Effect	**Damaging**	**Damaging**	Tolerated	**Damaging**
	Score	**0.036**	**0.001**	0.572	**0.002**
Mutation assessor.org	Effect	Low	**Medium**	Neutral	**Medium**
	FI score	1.895	**2.725**	−0.805	**2.89**
CONDEL	Effect	Neutral	**Deleterious**	Neutral	**Deleterious**
	Score	0.48	**0.59**	0.38	**0.65**
HSF3.0	Effect	**Potential new ESS, alter ESE**	**Alter donor splice site**	**Potential new donor site, new ESE/ESS**	No splice changes
NetGene2	Effect	No effect	**Eliminated donor site**	No effect	No effect

Results of mutation prediction models that assess the impact of missense mutations in the *CHM* gene. Predictions of non‐neutrality or change in protein function are highlighted in bold. PolyPhen‐2 HumVar Score – probabilistic classifier ranging from 0 (neutral) to 1 (damaging); SNAP Output – a neural‐network classifies mutations as neutral or nonneutral with a predicted accuracy value; pMUT Output – pathogenicity index ranging from 0 (neutral) to 1 (damaging). Scores >0.5 are pathological; PROVEAN Score – delta alignment score based on the reference and variant protein. Scores below a threshold at −2.5 are considered pathologenic SIFT Score – the scaled probability of whether substitutions are tolerated from 0 to 1. Scores <0.05 are pathologenic; mutationassessor.org FI Score – the predicted functional impact of substitutions. Scores above a threshold at 1.9 are considered pathological; CONDEL score – a weighted average of mutationassessor.org and FATHMM (Functional Analysis through Hidden Markov Models) ranging from 0 (neutral) to 1 (damaging). Scores >0.52 are pathological.

CHM reference sequences used: RAE1_HUMAN; NP_000381; ENSP00000350386.

ESS, exonic splicing silencer; ESE, exonic splicing enhancer.

The clinical characteristics of individuals with missense mutations are described in Table 5.

**Table 5:** Subjects with missense mutations

Subject	Mutation		Age (years)	Onset of symptoms (years)		Visual acuity (logMAR)		Visual field width	
	Nucleotide	Protein		Nyctalopia	Other symptoms	OD	OS	OD	OS
S1.1	c.1649T>C	p.L550P	44	10	20	0.9	2.3	5°	5°
S1.2	c.1649T>C	p.L550P	45	10	NC	0.7	0.5	NC	NC
S2.1	c.819G>T	p.Q273H	51	5	43	1	0.8	20°	10°
S2.2	c.819G>T	p.Q273H	22	NC	NC	0	0.18	135°	31°
S3.1	c.1370T>C	p.L457P	9	7	7	0	0	130°	130°
S3.2	c.1370T>C	p.L457P	17	15	15	0	0.1	140°	120°
S3.3	c.1370T>C	p.L457P	70	30	30	3	2.3	NC	NC
S4.1	c.238C>T; c.1327A>G	p.L80F; p.M443V	9	9	9	0	0	60°	60°

OD, right eye; OS, left eye; NC, not collected.

### Natural history

**Figure 3:**
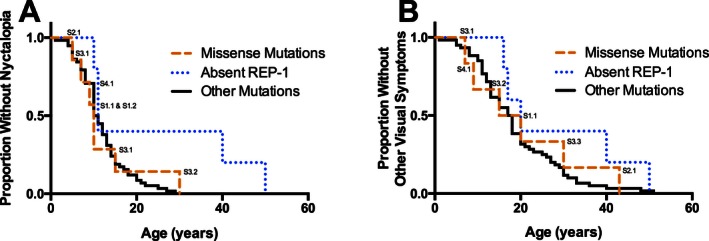
Kaplan–Meier survival curves demonstrating the self‐reported onset (in years) of nyctalopia (A) or other visual symptoms (B) in males affected by choroideremia. Subjects are grouped by the causative mutation: subjects with missense mutations (labeled; median age of onset of nyctalopia = 10 years, *n* = 7; median age of onset of other symptoms = 17.5 years, *n* = 6), subjects who do not express any Rab escort protein 1 due to whole gene deletions or deletions of the ATG start codon (median age of onset of nyctalopia = 11 years, *n* = 5; median age of onset of other symptoms = 20 years, *n* = 5), and subjects with other disease causing mutations (median age of onset of nyctalopia = 10.5 years, *n* = 58; onset of other symptoms = 17.5 years, *n* = 60). There is no significant difference between the survival curves of the three groups (nyctalopia: *P* = 0.08; other visual symptoms: *P* = 0.35).

The average onset of subjective symptoms of nyctalopia was at 12.6 ± 1.0 years of age (*n* = 71; mean ± SEM) and the average onset of other symptoms of visual loss (including loss of peripheral vision) was at 19.7 ± 1.3 years of age (*n* = 72), which agrees with previously published descriptions of CHM (MacDonald et al. 1993). The onset of nyctalopia in individuals harboring missense mutations (mean onset 12.3 ± 3.2 years, *n* = 7) was not statistically significantly different (Fig. 3; *P* = 0.08) from those who lacked any REP‐1 (mean onset 22.2 ± 7.4 years, *n* = 6) or other *CHM* mutations (mean onset 11.9 ± 0.8 years, *n* = 58). Likewise, there was no difference (*P* = 0.35) in the timing of onset of other visual symptoms for individuals harboring missense mutations (mean onset 20.7 ± 5.6 years, *n* = 6), individuals lacking any REP‐1 (mean onset 28.6 ± 6.9 years, *n* = 5), or individuals with other mutations (mean onset 18.9 ± 1.3 years, *n* = 61).

**Figure 4:**
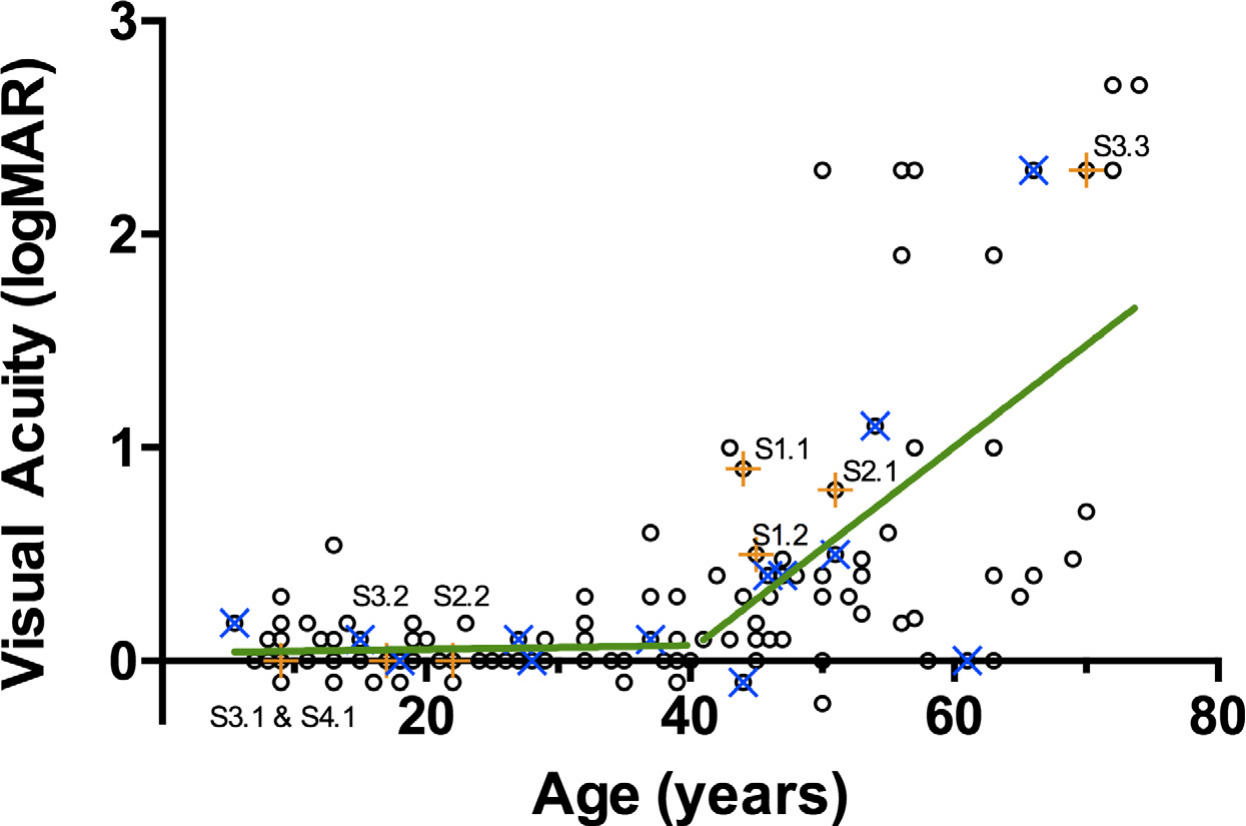
The best‐corrected visual acuity (logMAR equivalent) of affected males' better eye as a function of age (*n* = 128). The sample population is divided at the critical age (40 years old) into the ≤40 years old group or the >40 years old group. Individuals with missense mutations are indicated by an orange + and labeled; individuals who do not express any Rab escort protein 1 due to whole gene deletions or deletions of the ATG start codon are indicated by a blue ×. The rate of change of visual acuity predicted by a linear regression model (green line) in the ≤40 years old group is not significantly different from 0 (*P* = 0.71); the rate of change of visual acuity in the >40 years old group is 0.0483 logMAR units/year (*P* = 0.001).

The critical age for loss of VA in 128 males was 40 years of age, which defined the break in the biphasic model of the decline in visual acuity (Table 6; Fig. 4). VA was stable in males below 40 years of age (95% CI −0.004 to 0.006; mean = 0.001; *P* = 0.71), but declined at the rate of 0.048 logMAR units/year (95% CI 0.021–0.076; *P* = 0.001) after age 40. The cross‐sectional data were heteroskedastic and the decline in VA above 40 years of age varied dramatically between individuals. Individuals predicted to lack any *CHM* transcript or REP‐1 protein fragments (due to whole gene deletions or deletions spanning the ATG start codon on exon 1; *n* = 13) showed the same variability as those carrying other *CHM* mutations. Individuals harboring missense mutations in *CHM* (*n* = 8) demonstrated similar loss of VA to individuals with prematurely truncating mutations.

**Table 6:** Visual acuity of subjects grouped by severity

Age group (years)	1–10	11–20	21–30	31–40	41–50	51–60	61–70	71–80	Total
≤20/40 (≤0.3 logMAR)	10/10 (100%)	27/28 (96%)	17/17 (100%)	18/19 (95%)	13/24 (54%)	5/15 (33%)	3/12 (25%)	0/3	93/128 (73%)
>20/40 to ≤20/70 (>0.3 to ≤0.54 logMAR)	0/10	1/28 (4%)	0/17	1/19 (5%)	8/24 (33%)	3/15 (20%)	4/12 (33%)	0/3	17/128 (13%)
>20/70 to ≤20/200 (>0.54 to ≤1.0 logMAR)	0/10	0/28	0/17	0/19	2/24 (8%)	3/15 (20%)	2/12 (17%)	0/3	7/128 (5%)
>20/200 (>1.0 logMAR)	0/10	0/28	0/17	0/19	1/24 (4%)	4/15 (27%)	3/12 (25%)	3/3 (100%)	11/128 (9%)

Best‐corrected visual acuities (BCVAs) are in logMAR units. The BCVA of the better eye is used.

**Figure 5:**
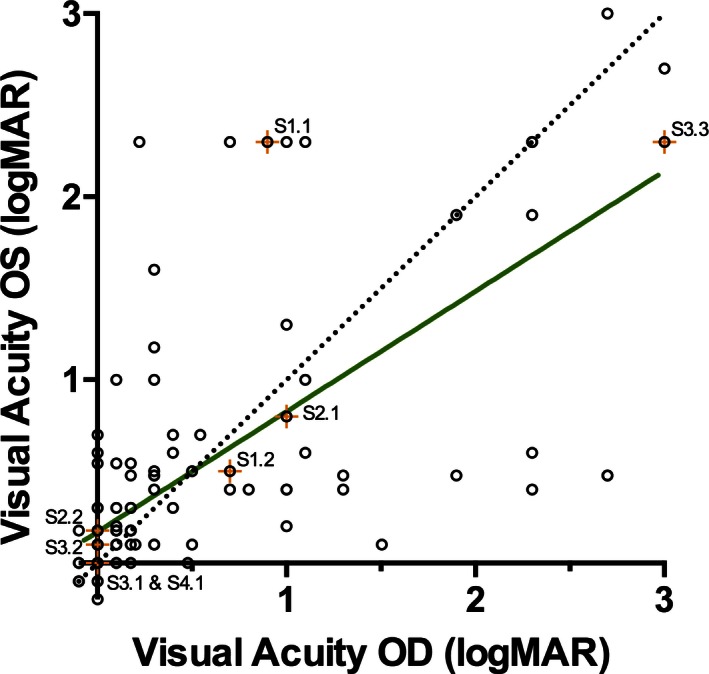
The intereye correlation of the best‐corrected visual acuities (logMAR) of affected males. A perfect correlation is also plotted for comparison (dotted line). The visual acuities of individuals' eyes are highly correlated (Spearman *r* = 0.76). Subjects with missense mutations are indicated by an orange + and labeled. OS, left eye; OD, right eye

There was a strong correlation (Spearman *r* = 0.76) between the VA of both eyes in affected males (Fig. 5). However, these data were heteroskedastic, with higher variability at lower VA. Very poor VA in one eye (worse than 1.0 logMAR) did not necessarily correspond with similar poor acuity in the fellow eye.

**Figure 6:**
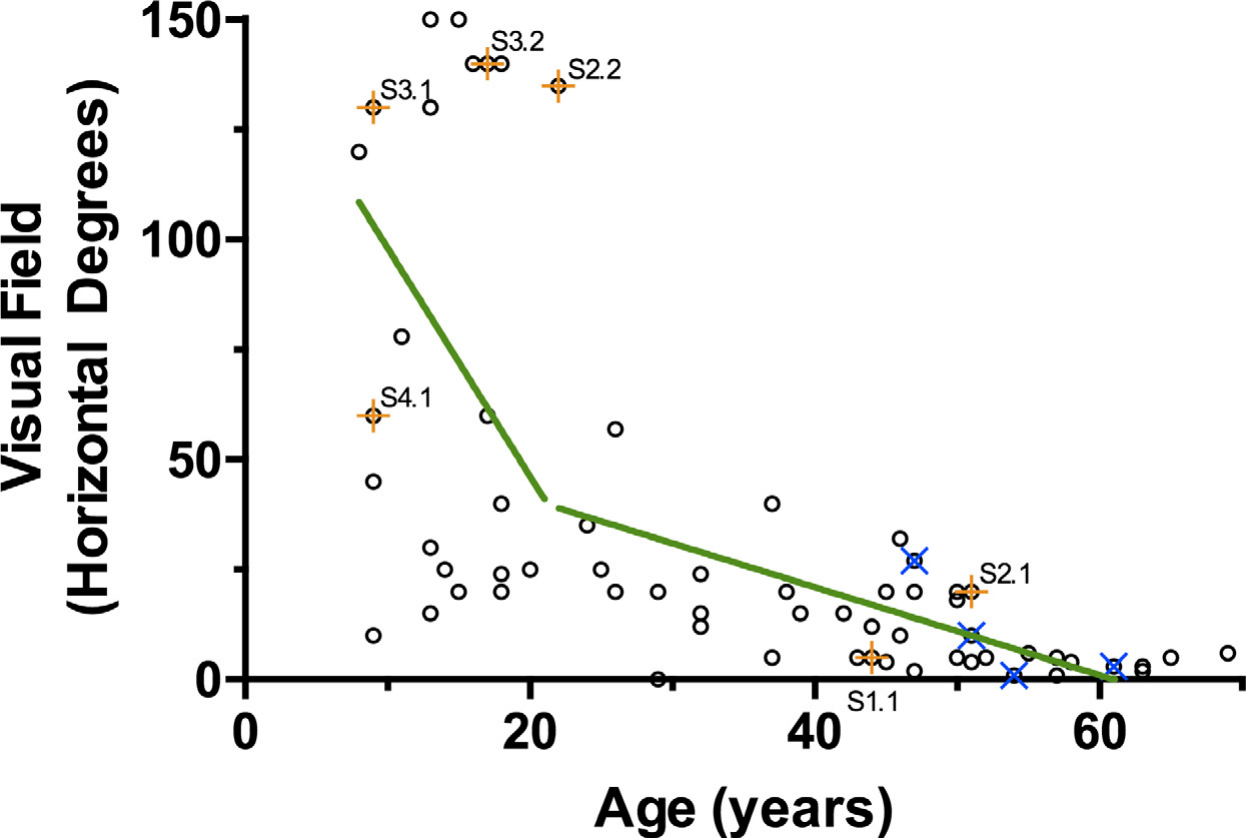
The visual field (the continuous visual field across the horizontal meridian, in degrees) of affected males' better eye as a function of age (*n* = 64). The sample population was divided at the critical age (20 years old), separating individuals into the ≤20 years old group and the >20 years old group. Individuals with missense mutations are indicated by an orange + and labeled; individuals who do not express any Rab escort protein 1 due to whole gene deletions or deletions of the ATG start codon are indicated by a blue ×. In a linear regression model (green line), age was not a significant predictor of visual field in the ≤20 years old group (*P* = 0.785); the rate of change of visual fields in the >20 years old group is a loss of 0.868 horizontal degrees per year (*P* = 0.005).

**Figure 7:**
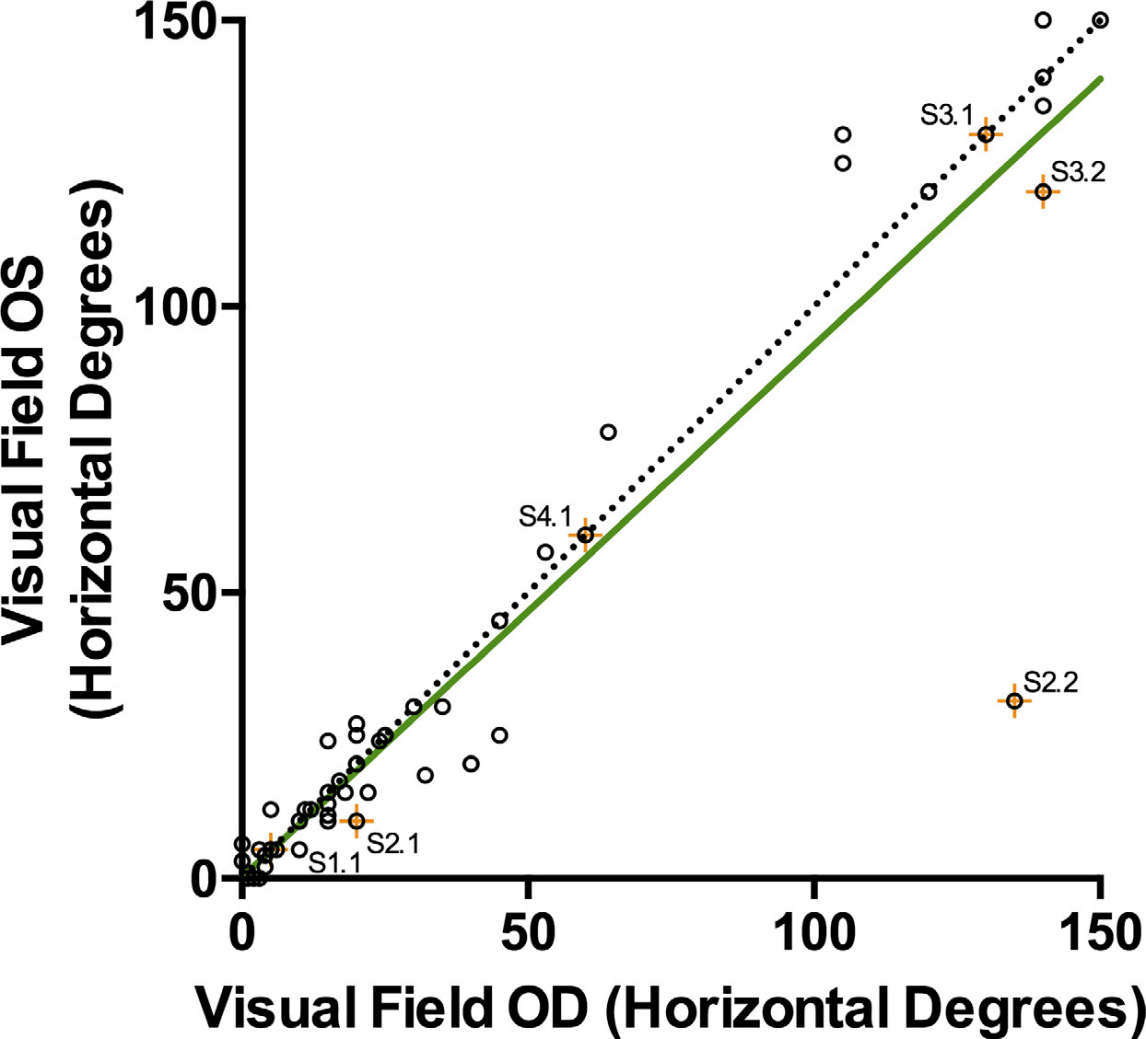
The intereye correlation of the visual fields (horizontal degrees across the meridian) of affected males. A perfect correlation is plotted for comparison (dotted line). The visual fields of individuals' eyes are very highly correlated (Spearman *r* = 0.95). Subjects with missense mutations are indicated by an orange + and labeled. OS, left eye; OD, right eye.

The critical age for the rate of decline in VFs in 64 males was 20 years of age (Fig. 6). The VFs of individuals less than 20 years of age were highly variable and the rate of change was not significant (95% CI 7.42 to −9.58 horizontal degrees/year; mean = −1.079; *P* = 0.79). Above the critical age of 20, the width of visual fields declined by 0.87 horizontal degrees/year (95% CI −0.28 to −1.46; *P* = 0.006). Individuals who were predicted to lack any *CHM* transcript or REP‐1 protein fragments (due to whole gene deletions or deletions spanning the ATG start codon on exon 1; *n* = 5) showed the same variability as those with other *CHM* mutations. There was also no difference in the rate of decline in individuals with missense *CHM* mutations (*n* = 6). There was a very high correlation (Spearman *r* = 0.95) between the VF of both eyes in affected males (Fig. 7). The correlation was consistent in both the early and late stages of CHM.

The ERG data and fundus changes collected in the databases did not contribute further to characterizing the disease states as nearly all individuals exhibited decreased ERG amplitudes for both rod and cone‐driven responses and stereotypical fundus changes (data not shown). The variability between testing sites for ERG (without lab‐specific normal values included in submitted data for comparison) as well as the variability of the response precluded any further analysis of the ERG.

## Discussion

This study compared the genotypes and phenotypes of affected males with confirmed *CHM* mutations and facilitated the evaluation of the clinical course of CHM within the context of affected individuals' genotypes. Our sample population showed frequencies of mutations similar to those documented in a public *CHM* mutation database indicating that our sample was representative of the CHM population (www.lovd.nl). In comparison to the HGMD, for this study of 128 CHM males, missense mutations in the *CHM* gene were uncommon (4%) and deletions, nonsense, and splice mutations were predominant. Although there were a few mutations shared by families who were not known to be related, the vast majority of mutations were unique. We found that males with missense mutations in the *CHM* gene did not have a different natural history than males with effective “null” mutations (nonsense, deletion, or splice site). These results suggest that there are no genotype–phenotype correlations in males affected by CHM.

The novel missense mutations identified in this study may provide further insight into the function of REP‐1 protein as very few missense mutations have been identified in CHM. Protein expression and prenylation activity assays will be important to confirm the *in silico* predictions on the effect of the single amino acid substitutions (i.e., lack of REP‐1 expression with the p.Gln273His or p.Leu457Pro substitutions). The p.Leu80Phe and p.Met443Val substitutions were predicted to be less damaging by modeling programs when either substitution was considered individually. However, when assessing both of these mutations together using homology modeling based on predicted protein structures (which is more likely to represent the mutations' true effect *in vivo*), REP‐1 was predicted to be unstable. Protein assays and functional assays of prenylation will be particularly important to confirm that these mutations are pathogenic. Alternatively, a third mutation deep within the intron sequences, which are not sequenced, or in a remote regulatory sequence could also affect the *CHM* phenotype, if the p.Leu80Phe and p.Met443Val variants are not disease causing. While the possibility of a dominant‐negative effect of these missense mutations cannot be ruled out in this retrospective study with our *in silico* analysis, the absence of any female carriers of missense mutations reporting CHM phenotypes in our databases (data not shown) suggests that female carriers are haplosufficient and there are no dominant‐negative effects of the REP‐1 with missense mutations.

This study also showed that there were no apparent genotype–phenotype correlations, within the spectrum of *CHM* mutations that were found, with regard to onset of symptoms, and the decline in BCVA or visual fields. The absence of genotype–phenotype correlations is consistent with the near universal lack of REP‐1 protein expression in CHM, regardless of whether the causative mutation is a missense mutation; partial or whole gene deletion; or a prematurely truncating nonsense or splice site mutation (MacDonald et al. 1998; Beaufrere et al. 1999; Sergeev et al. 2009; Esposito et al. 2011). The phenotypic variability in CHM may result from a combination of other less understood contributors, such as environmental factors (e.g., ultraviolet light exposure, [Glickman 2011] nutrition, [Duncan et al. 2002] concomitant statin use, [Abstracts of the 50th ISCEV (International Society for Clinical Electrophysiology of Vision) International Symposium, June 3–7, 2012, Valencia, Spain, 2012; Zhou et al. 2013], etc.), other genetic factors (e.g., epistatic effects from polymorphisms in other genes/proteins associated with REP‐1 prenylation, such as Rab escort protein‐2 [Cremers et al. 1994]) or stochastic effects.

Previous studies of BCVA in CHM have described the rate of change in a linear fashion across the lifespan of affected males ranging from 0.0072 to 0.018 logMAR units/year (Roberts et al. 2002; Coussa et al. 2012). From our analysis, however, and in keeping with the relative preservation of the fovea until late in the course of CHM, a two‐phase model can also be considered: a quiescent period of maintained BCVA followed by a phase with progressive loss of central acuity. This model was first used by Coussa et al. (2012) who calculated the rate of change above 50 years of age to be 0.0206 logMAR units/year. Using similar methods, we determined that the critical age to separate the two phases of degeneration was earlier, at age 40. Further, despite using more conservative logMAR estimates of semiquantitative measures (e.g., our study considered counting fingers to be 1.9 logMAR, not 2.6 logMAR) and having a younger critical age of 40 years (which biases our model toward underestimating the rate of change), we calculated that BCVA would decline at a faster rate of 0.048 logMAR units/year above the age of 40. Finally, despite the variability in the decline of BCVA that may occur within and among individuals, BCVA will still be an important safety measure for the evaluation of any adverse events.

From our observations, a two‐phase model also described the progression of visual fields in CHM, with the critical age defined as age 20. Below age 20, the width of visual fields was highly variable, which may have reflected either variations in the disease itself or issues of compliance with perimetry testing in children or teenagers. The critical age of 20 years is consistent with patients' self‐reported onset of symptoms related to constriction of their visual fields. Above age 20, the width of the visual fields declined at a linear rate of 0.87 horizontal degrees/year. The width of the continuous horizontal meridian provides a metric for quantifying Goldmann VF results, although surviving photoreceptor and retinal pigment epithelium (and resulting VF) islands have irregular borders. No data exist on the repeatability of VF in CHM, but in retinitis pigmentosa, the within‐visit and intervisit variability in the measured diameter of the Goldmann VF is 17–22% with V4e or III4e isopters (Berson et al. 1985; Bittner et al. 2011). The variability in retinitis pigmentosa was consistent for VF larger than 14 degrees in diameter, but if VF was less than 14 degrees in diameter, the variability increased significantly (Bittner et al. 2011). Assuming that Goldmann VFs of patients with CHM exhibit the same variability as those with retinitis pigmentosa, the time to reach a significantly different VF from baseline would range from 9.6 years in a 20‐year‐old male with CHM (predicted to have a baseline VF width of 38°) to 5.2 years in a 40‐year‐old (predicted to have a baseline VF width of 20°). The decline in VF is also much more symmetric than BCVA in advanced CHM; thus, comparison of VF between treated and untreated fellow eyes may allow for better evaluation of treatment efficacy.

The University of Alberta database and eyeGENE^™^ database provided approximately equal proportions of subject data (46% and 54%, respectively). The data records within the University of Alberta database were a combination of patients assessed at the University of Alberta by the author (IMM) and clinical data submitted to the University of Alberta for *CHM* genotyping. Thus, the interpretation of the clinical data (i.e., measuring the width of the visual field) collected at the University of Alberta could be verified by the authors; the submitted data from external ophthalmologists were also verified when raw data were included. The eyeGENE^™^ database provides data within a standardized format, with the results interpreted by the submitted ophthalmologist (i.e.*,* measured width of the visual field or ERG waveform amplitudes and implicit times). The authors were unable to verify the consistency of the interpretations of the submitted data. The clinical data was submitted to eyeGENE^™^ as part of their regular ophthalmic care, with visual fields and electroretinograms done at the discretion of their treating ophthalmologist as part of the diagnosis or routine follow‐up.

This study defines the natural history changes in CHM, describing the onset of symptoms as well as the rate of change in BCVA and VF. BCVA variably declines above the age of 40, whereas the width of VFs constrict at a relatively uniform and predictable rate. Furthermore, the intereye correlation of VFs is greater than BCVA in advanced CHM. No genotype–phenotype correlations were identified in this study: missense mutations did not cause a milder phenotype than whole gene deletions or other *CHM* mutations. Thus, the inclusion of individuals with missense mutations would not affect the length of follow‐up required in clinical trials as their rate of disease progression is similar to individuals with other *CHM* mutations. In conclusion, this study does not support any specific exclusion criteria for CHM patients from clinical trials due to their specific genotypes as no genotype–phenotype correlations could be identified.

## Conflict of Interest

The authors have no proprietary or commercial interest in any materials discussed in this article.

## Supporting information


**Data S1.** *In silico* mutation analysis programs used in this study.
