# Supplementary material for: Analysis of a large choroideremia dataset does not suggest a preference for inclusion of certain genotypes in future trials of gene therapy
Source: Mol Genet Genomic Med. 2016 Feb 28;4(3):344–58. doi: 10.1002/mgg3.208 (PMC4867567; doi:10.1002/mgg3.208)
Supplement: Supplementary file 1 — Data S1. In silico mutation analysis programs used in this study. [file MGG3-4-344-s001.docx]

Supporting Information:

*In silico* mutation analysis programs used in this study. All programs were accessed May 2014.

1. Polyphen-2 HumVar. Available at: <http://genetics.bwh.harvard.edu/pph2/> (Adzhubei et al., 2010)
2. SNAP. Available at: <https://www.rostlab.org/services/snap/> (Bromberg and Rost, 2007)
3. pMUT. Available at: <http://mmb.irbbarcelona.org/PMut/> (Ferrer-Costa et al., 2005)
4. PROVEAN. Available at: <http://provean.jcvi.org/> (Choi et al., 2012)
5. SIFT. Available at: <http://sift.jcvi.org/> (Ng and Henikoff, 2001)
6. MutationAssessor. Available at: <http://mutationassessor.org/>
7. CONDEL. Available at: <http://bg.upf.edu/fannsdb/> (Gonzalez-Perez and Lopez-Bigas, 2011).
8. Human Splice Finder-3. Available at: <http://www.umd.be/HSF3/> (Desmet et al., 2009)
9. NetGene2 programs. Available at: <http://www.cbs.dtu.dk/services/NetGene2/> (Brunak et al., 1991).
